# Two-Eyed Seeing and artificial intelligence: Enhancing healthcare delivery in Indigenous communities requires an ethical and culturally relevant public health framework

**DOI:** 10.17269/s41997-025-01037-1

**Published:** 2025-04-23

**Authors:** Amal Khan, Veronica McKinney, Ivar Mendez

**Affiliations:** 1 Department of Community Health and Epidemiology, University of Saskatchewan, Saskatoon, SK Canada; 2 Virtual Health Hub, Saskatoon, SK Canada; 3 Northern Medical Services, College of Medicine, University of Saskatchewan, Saskatoon, SK Canada; 4 Department of Surgery, University of Saskatchewan, Saskatoon, SK Canada

**Keywords:** Two-Eyed Seeing, Indigenous data sovereignty, Culturally safe AI, Ethical AI implementation, Public health framework, Co-governance in AI design, Vision à deux yeux, Souveraineté des données autochtones, IA culturellement sûre, Application éthique de l’IA, Cadre de santé publique, Gouvernance conjointe de la conception de l’IA

## Abstract

Artificial intelligence (AI) is poised to transform healthcare delivery; this may be particularly important to underserved rural, remote, and Indigenous communities. This commentary explores the potential of AI to enhance healthcare access and outcomes of these populations while emphasizing the need for culturally safe and ethical implementation. By integrating AI with Indigenous knowledge systems through the Two-Eyed Seeing approach, we propose a framework that ensures that AI-driven healthcare is equitable, culturally sensitive, and effective. This public health perspective highlights the importance of approaching AI advancements with a culturally appropriate and relevant lens.

## Résumé

L’intelligence artificielle (IA) est sur le point de transformer la prestation des soins de santé, ce qui pourrait être particulièrement important pour les communautés autochtones, rurales et éloignées mal desservies. Dans ce commentaire, nous explorons la possibilité que l’IA améliore l’accès aux soins de santé et les résultats cliniques de ces populations, mais nous soulignons la nécessité de l’appliquer de façon éthique et culturellement sûre. En intégrant l’IA aux systèmes de savoir autochtones selon l’approche de la «vision à deux yeux», nous proposons un cadre pour que les soins de santé guidés par l’IA soient équitables, culturellement appropriés et efficaces. Cette perspective de santé publique fait ressortir l’importance d’aborder les avancées en IA selon un prisme pertinent et culturellement approprié.

## Introduction

The integration of artificial intelligence (AI) into healthcare systems presents an unprecedented opportunity to enhance service delivery, particularly for underserved populations. AI offers the potential to improve clinical decision-making, optimize workflows, reduce costs, and improve outcomes, making healthcare more efficient and accessible (Maleki Varnosfaderani & Forouzanfar, 2024; Yelne et al., 2023). Efficiency does not necessarily equate with equitability, and access is more than having healthcare options present. Therefore, the deployment of AI in healthcare must be approached with consideration and incorporation of Indigenous approaches to health and wellness, along with the recognition that operationalization will require attention to the complex systems involved when applied to Indigenous and other marginalized communities. Historical and intergenerational impacts of colonialism and systemic racism in healthcare have profoundly shaped Indigenous perspectives on technology, including AI, often fostering skepticism and mistrust toward healthcare innovations. The long history of data extraction and misuse has reinforced these concerns, as data mining practices have perpetuated colonial structures, highlighting the need for Indigenous data sovereignty (Roberts & Montoya, 2023). Additionally, the collective memory of systemic racism and community trauma continues to shape trust in AI, as past discriminatory healthcare practices have led to caution regarding the role of algorithms in decision-making and health governance (Kuhlberg et al., 2023). This commentary advocates for a public health perspective based on the Two-Eyed Seeing approach (Bartlett et al., 2012) that prioritizes cultural safety, equity, and ethical considerations in the implementation of AI-driven healthcare.

## AI in healthcare: A tool for transformation

AI has already demonstrated significant potential across various aspects of healthcare (Shortliffe & Sepúlveda, 2018). For instance, AI-assisted diagnostic tools have shown high accuracy in detecting diseases such as cancer (Liao et al., 2022), while precision medicine applications enable personalized treatment and management plans based on patients’ genetic profiles (Johnson et al., 2021). Such advancements can revolutionize healthcare by improving patient outcomes and addressing workforce shortages through automation (Topol, 2019). However, the deployment of AI in healthcare, particularly in rural and Indigenous communities, requires careful consideration of cultural and contextual factors. AI-driven technologies must be adapted to meet the specific needs of these populations, ensuring that they do not exacerbate existing health disparities but instead contribute to their reduction (World Health Organization, 2021).

## Integrating Indigenous knowledge: The Two-Eyed Seeing approach

A key consideration in the deployment of AI-driven healthcare in Indigenous communities is the integration of Indigenous knowledge systems. The Two-Eyed Seeing approach, as articulated by Mi'kmaq Elder Albert Marshall, provides a valuable framework for this integration. This approach advocates for the use of both Indigenous and Western perspectives to create a more holistic and inclusive healthcare model (Bartlett et al., 2012).

In practice, the Two-Eyed Seeing approach would involve co-designing AI systems with Indigenous communities, scholars, leadership, and front-line Indigenous providers, especially those ensconced in the culture and traditions of the communities they serve, ensuring that these technologies are not only technically effective but also culturally safe (Government of Canada, 2023). Indigenous perspectives differ from Western ideologies in their holistic approach, emphasizing interconnectedness between health, land, and community rather than viewing healthcare as an isolated system. Unlike Western frameworks that prioritize empirical quantification, Indigenous knowledge integrates lived experience, oral traditions, and relational accountability (Nakashima & Roué, 2002). This could include incorporating Indigenous ways of knowing and doing into AI algorithms, which would enhance the cultural relevance of healthcare services delivered through virtual care platforms. Such an approach would ensure that AI-driven healthcare is responsive to the unique needs and values of Indigenous populations, fostering greater acceptance and utilization. Further, it is critical to avoid replication of existing systemic approaches that have been harmful to many Indigenous populations. It is also important to recognize that the term Indigenous encompasses a large diversity of people across vast geographical areas. This means that while core concepts may be similar, there is an opportunity to build a system that is attentive to unique aspects of communities previously not addressed but significant to the population served.

## Culturally safe healthcare through AI

AI has the potential to support culturally safe healthcare practices by delivering personalized interventions that respect the cultural values of these diverse Indigenous communities (Silano, 2024). For example, AI-driven virtual health assistants can be designed to provide advice and support that aligns with traditional practices and beliefs, enhancing the cultural appropriateness of care. Moreover, AI can facilitate the ethical collection and analysis of health data, ensuring that Indigenous communities maintain sovereignty over their information. Implementing frameworks such as the CARE Principles (collective benefit, authority to control, responsibility, and ethics) ensures that AI systems operate under guidelines that prioritize community interests and governance (Carroll et al., 2020). For instance, Indigenous-led AI projects, like Te Hiku Media’s development of a Māori language model, an automatic speech recognition (ASR) model, exemplify successful integration of ethical data collection by ensuring that data remain under Indigenous ownership and control (TIME, 2024). Such approaches aid in ensuring that the collection and analysis of these data is in line with Indigenous approaches as well as the needs of the community. By prioritizing community control and data governance, AI technologies can build trust and ensure that they serve the interests of Indigenous populations rather than imposing external solutions (Cordes et al., 2024; University of British Columbia, Faculty of Arts, 2024).

## The need for an ethical public health framework

As AI technologies continue to evolve, addressing the ethical challenges associated with their implementation in healthcare is critical. This includes ensuring transparency, accountability, and the mitigation of potential biases in AI systems. A public health framework that integrates the principles of equity, accessibility, and cultural safety is essential for guiding the ethical deployment of AI in healthcare (World Health Organization, 2021). Specific ethical challenges include but are not limited to biases in datasets, where underrepresentation of certain groups can lead to AI systems that perform poorly for those populations, potentially exacerbating health disparities. For instance, AI algorithms trained predominantly on data from high-income countries may not generalize well to underrepresented populations, leading to disparities in healthcare outcomes (Kaushal et al., 2020). Additionally, biased healthcare algorithms can require racial or ethnic minorities to be more ill than their white counterparts to receive the same level of care, thereby amplifying existing inequalities (Obermeyer et al., 2019). These examples underscore the necessity for diverse and representative datasets in AI development to ensure equitable healthcare delivery. In Canada, legislative initiatives such as the Digital Charter Implementation Act (Bill C-27) aim to address these challenges by establishing frameworks for AI governance and data protection (Government of Canada, 2022).

A framework that involves the co-design of AI programs and systems with Indigenous communities, incorporating Indigenous scholars and leaders, may provide a way to ensure that these technologies and their processes are aligned with Indigenous cultural values and data sovereignty (First Nations Information Governance Centre, 2020) and are grounded in their healthcare needs. Continuous monitoring and evaluation of AI-driven healthcare programs are also necessary to assess their impact and ensure they deliver the intended benefits without unintended consequences. This could be achieved through mechanisms such as government oversight committees, public health agencies, and partnerships with Indigenous organizations, ensuring that monitoring processes are collaborative and culturally informed (Kansas Health Institute, 2025).

## Conclusion

Applying AI to healthcare delivery presents considerable opportunities to enhance health outcomes, especially in the most underserved populations, such as Indigenous communities. However, achieving this potential requires an approach that emphasizes cultural safety, fairness, and ethical principles. By embracing the Two-Eyed Seeing philosophy and incorporating Indigenous knowledge systems in designing AI frameworks, we can ensure that AI technologies are implemented in a manner that is culturally relevant and responds to Indigenous communities’ unique needs, recognizing that the replication of current systems will simply perpetuate ongoing harm. The success of Te Hiku Media’s Māori language AI model demonstrates how Indigenous data sovereignty and ethical co-governance can empower Indigenous communities, ensuring AI serves their cultural and linguistic needs. Conversely, failures in predictive policing AI have shown how biases embedded in algorithms disproportionately harm marginalized populations when Indigenous perspectives are excluded (Benjamin, 2020). This strategy may not only improve the impact of AI in healthcare delivery to Indigenous populations but also support the overarching aim of promoting health equity for all.

## Data Availability

Not applicable.
